# DESCRIBING HOW ADAPTIVE LEADERSHIP IN LONG-TERM CARE CAN MOTIVATE AND PREPARE CARE WORKERS TO PROMOTE QUALITY

**DOI:** 10.1093/geroni/igae098.2793

**Published:** 2024-12-31

**Authors:** Kirsty Haunch, Reena Devi, Karen Spilsbury

**Affiliations:** University of Leeds, Leeds, England, United Kingdom; University of Leeds, Leeds, England, United Kingdom; University of Leeds, Leeds, England, United Kingdom

## Abstract

Adaptive leadership is one approach to enhance the experience of people living and working in long-term care (LTC) residential environments. Adaptive leadership is comprised of a set of strategies aimed at enabling care teams to work together, or create organisational capacity to support culture changes, to accomplish person-centred environments. The defining feature of adaptive leadership is the separation of technical solutions (i.e., applying existing knowledge/techniques to problems) and adaptive solutions (i.e., a shift in how people work together not just what they do). Our aim is to describe and illustrate adaptive leadership behaviours in LTC and the potential benefit for the processes and outcomes of care. We conducted a documentary analysis of (publicly available) UK regulatory inspection reports. Twenty reports, judged as providing outstanding quality were purposively sampled to represent variations in ownership, size, and geographical location. Data were initially analysed using content analysis. Heitz’s adaptive leadership framework was used to frame the data. Adaptive leadership behaviours helped to engage, empower, and energise the workforce to accomplish meaningful change. These behaviours included accepting everyone’s unique perspective on how to solve problems, a willingness to learn lessons, an openness to feedback, embrace diversity, a fair and inclusive workplace, continuous growth, innovation and emotional intelligence. Key structural components enabling adaptive leadership included organisational support, ‘in house’ operational support, peer support and training. Our work highlights the benefit of recognising adaptive challenges in LTC and empowering teams to apply the most appropriate problem-solving solutions, and in particular applying adaptive solutions when merited.

